# Epidemiology of HIV among female sex workers, their clients, men who have sex with men and people who inject drugs in West and Central Africa

**DOI:** 10.7448/IAS.16.4.18751

**Published:** 2013-12-02

**Authors:** Erin Papworth, Nuha Ceesay, Louis An, Marguerite Thiam-Niangoin, Odette Ky-Zerbo, Claire Holland, Fatou Maria Dramé, Ashley Grosso, Daouda Diouf, Stefan D Baral

**Affiliations:** 1 Department of Epidemiology, Johns Hopkins Bloomberg School of Public Health Baltimore, MD, USA; 2 Joint United Nations Programme on HIV/AIDS, Banjul, Gambia; 3 Programme de Lutte Contre le SIDA Chez Les Populations Hautement Vulnérables (PLS-PHV) Ministère de la Sante et de la lutte contre le SIDA, Abidjan, Côte d'Ivoire; 4 Programme d'Appui au Monde Associatif et Communautaire (PAMAC), Ouagadougou Burkina Faso; 5 Enda Santé, Dakar, Senegal

**Keywords:** men who have sex with men, sex work, people who inject drugs, HIV epidemiology, West Africa, Central Africa, prevalence, risk factors

## Abstract

**Introduction:**

The West and Central Africa (WCA) sub-region is the most populous region of sub-Saharan Africa (SSA), with an estimated population of 356 million living in 24 countries. The HIV epidemic in WCA appears to have distinct dynamics compared to the rest of SSA, being more concentrated among key populations such as female sex workers (FSWs), men who have sex with men (MSM), people who inject drugs (PWID) and clients of FSWs. To explore the epidemiology of HIV in the region, a systematic review of HIV literature among key populations in WCA was conducted since the onset of the HIV epidemic.

**Methods:**

We searched the databases PubMed, CINAHL and others for peer-reviewed articles regarding FSWs, MSM and PWID in 24 countries with no date restriction. Inclusion criteria were sensitive and focused on inclusion of any HIV prevalence data among key populations. HIV prevalence was pooled, and in each country key themes were extracted from the literature.

**Results:**

The search generated 885 titles, 214 abstracts and 122 full articles, of which 76 met inclusion and exclusion criteria providing HIV prevalence data. There were 60 articles characterizing the burden of disease among FSWs, eight for their clients, one for both, six for MSM and one for PWID. The pooled HIV prevalence among FSWs was 34.9% (*n=*14,388/41,270), among their clients was 7.3% (*n=*435/5986), among MSM was 17.7% (*n=*656/3714) and among PWID from one study in Nigeria was 3.8% (*n=*56/1459).

**Conclusions:**

The disproportionate burden of HIV among FSWs appears to be consistent from the beginning of the HIV epidemic in WCA. While there are less data for other key populations such as clients of FSWs and MSM, the prevalence of HIV is higher among these men compared to other men in the region. There have been sporadic reports among PWID, but limited research on the burden of HIV among these men and women. These data affirm that the HIV epidemic in WCA appears to be far more concentrated among key populations than the epidemics in Southern and Eastern Africa. Evidence-based HIV prevention, treatment and care programmes in WCA should focus on engaging populations with the greatest burden of disease in the continuum of HIV care.

## Introduction

The sub-region of West and Central Africa (WCA) is the most populous of sub-Saharan Africa (SSA), with a combined population of roughly 356 million [1]. The region possesses a distinct cultural, economic and historical diversity. The majority of countries purport French as their national language, while English is the state language for four countries, and Spanish and Portuguese are both spoken within the region. Fifteen of the countries in WCA are classified by the World Bank Atlas method as low income (>US$1025), including Benin, Burkina Faso, Cape Verde, Central African Republic, Chad, the Democratic Republic of Congo (DRC), the Gambia, Guinea, Guinea-Bissau, Liberia, Mali, Mauritania, Niger, Sierra Leone and Togo [2]. Côte d'Ivoire, Cameroon, Ghana, Nigeria, the Republic of Congo, Senegal and São Tomé and Príncipe are categorized as low-middle income (US$1026 to US$4035) [2]. One country in the region is upper-middle income (Gabon), and one is ranked as a high-income country (Equatorial Guinea), mainly due to newly found oil reserves and a population under 1 million [2].

**Figure 1:**
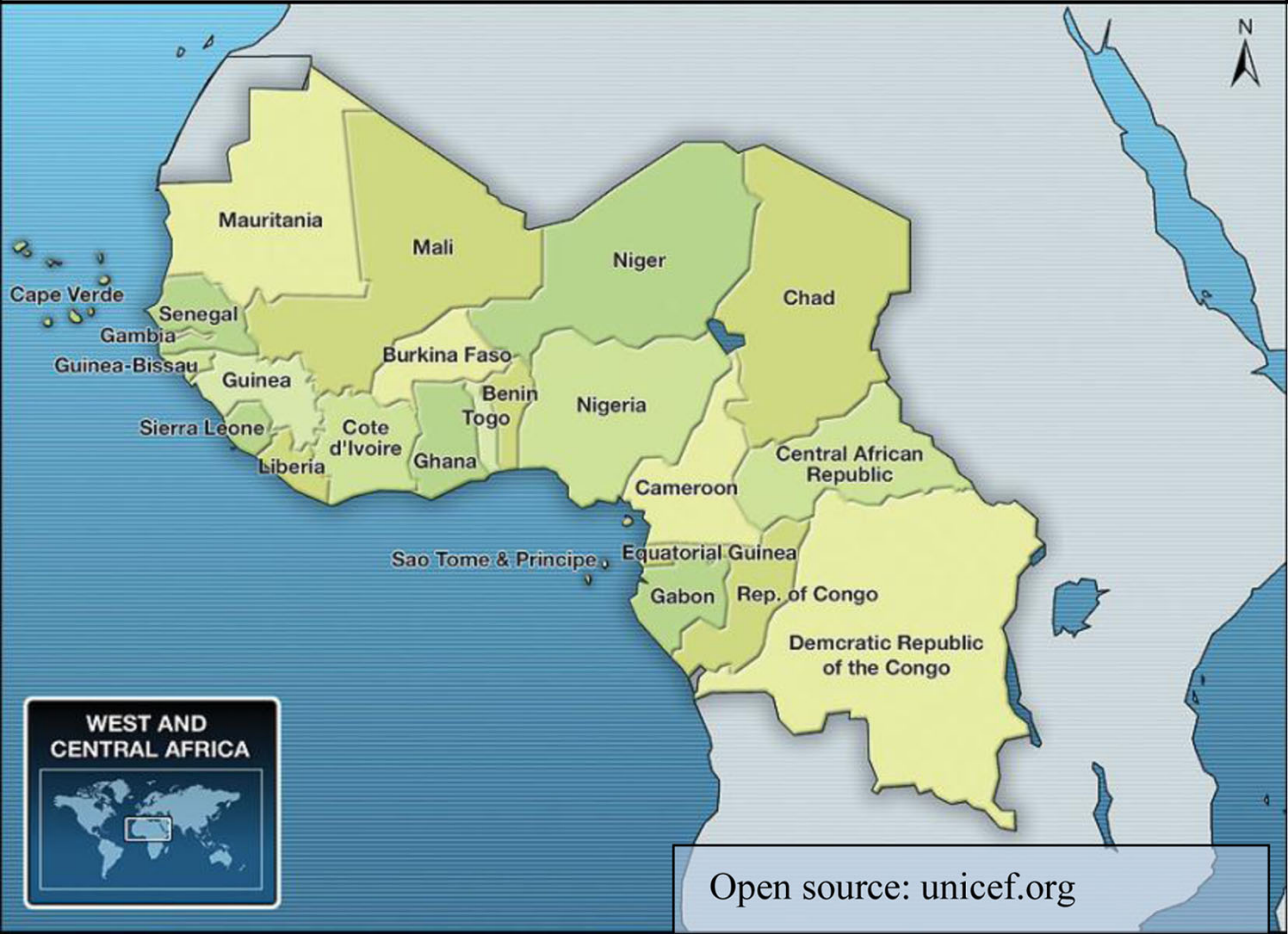
Map of West and Central Africa.

Historically and economically multifarious, the region has not been immune to the HIV epidemic. The first reported cases of HIV emerged in the mid-1980s, and national surveillance bodies such as National AIDS Committees (NACs) were established over the subsequent decade [3]. Early phylogenetic subtyping revealed unique regional dynamics, with both HIV-1 and HIV-2 circulating, and the majority of global cases of HIV-2 found in West Africa. Concurrently, the origins and greatest subtype diversity of HIV-1 were reported in Central Africa [4] (Figure 1).

Nevertheless, regional epidemiological reporting has traditionally been immersed in the overall context of SSA. Trends in the HIV epidemic show that SSA possesses the highest burden of HIV, and 69% of the global population of people living with HIV reside within its borders [23.5 million (22.1–24.8 million)] [5, 6]. While these statistics show an important burden of disease on the continent, they mask disparities in HIV epidemics regionally [7]. Countries in East and South Africa report consistently generalized epidemics among reproductive-age adults (ages 15–49), which is defined through the Joint United Nations Programme on HIV/AIDS (UNAIDS) criteria as HIV prevalence consistently higher than 1% in antenatal clinics [8, 9]. Nine out of the 15 Southern African Development Community (SADC) members report national prevalence over 10% [5, 6, 10]. Reproductive-age adult estimates are as high as 25.9% in Swaziland and 24.8% in Botswana [11]. Comparatively, national prevalence in WCA has remained low or moderate since HIV surveillance reporting began, with current general-population estimates ranging from 0.02 to 4.5% [5, 6, 12]. Twelve countries in the sub-region report national prevalence under 2% [5]. Consequently, the majority of these countries’ HIV epidemics are classified as mixed, concentrated or borderline generalized [6, 12].

The international community has recently noted that classifications of the HIV epidemic based on prevalence data often limit understanding of the complexity of transmission and appropriate prevention strategies. However, concentrated epidemics have historically been defined as occurring in countries where HIV prevalence is consistently higher than 5% in at least one subgroup within the population, but less than 1% in antenatal clinics [7, 9]. These subgroups are generally considered to be female sex workers (FSWs), men who have sex with men (MSM) and people who inject drugs (PWID) [7, 13]. There is less clarity around mixed epidemics, although these are generally agreed to be low-level generalized epidemics ranging from 2 to 5% HIV prevalence in the general population, and high transmission rates in subgroups of the population [7]. Based on this, the HIV epidemics in countries in WCA are predominantly mixed or concentrated.

Researchers have suggested that the complexity of the regional dynamics in WCA has not been dissected adequately [12, 14–16]. Underlying drivers such as migration patterns, subtype diversity, significant regional variations of the disease and at-risk populations are understudied [11, 12, 16–19]. In an era where the global spread of HIV is on the decline, data are progressively emerging to show sustained or expanding transmission in populations at high-risk for HIV [15, 20–22]. However, national surveillance systems, particularly in low and middle-income countries, remain constructed on population-level studies such as the Demographic and Health Survey and antenatal care surveillance data [6, 13]. These methods provide a global overview of basic risk factors associated with transmission, but they do not capture data characterizing sex work and other transactional or compensated sex, same-sex practices and drug use outside of alcohol consumption, all of which are demonstrated high-risk factors and contributors to the acquisition and transmission of HIV [11, 21, 23].

Globally, surveillance shows that groups such as FSWs, their clients, MSM and PWID sustain a higher burden of disease in concentrated epidemics and substantially contribute to new infections annually [4, 7, 18, 22, 24]. In settings such as Southeast Asia and Latin America, general-population HIV prevalence remains similar to that of WCA, and a higher burden of disease is observed among key populations. For example, Pakistan and Indonesia report 25% and 35% prevalence among PWID, respectively [25]. Vietnam and Chile report an HIV prevalence rate of 15% and 20% among MSM, respectively [25, 26]. Myanmar (Burma) reports a prevalence of 10% among FSWs, and Brazil reports 4.9% [25, 26]. All of these reported levels are roughly five to thirty times higher than general-population prevalence in the specific countries listed [25, 26]. National-level responses on these continents have included programmes for key populations, and noteworthy advances in the reduction of new infections have been reported over time [27, 28]. In contrast, WCA reports partial or sporadic data for key populations and limited government-level policies defining key population treatment and prevention needs [5]. National surveillance and programming in WCA subsequently remain rooted in broad HIV prevention messaging and approaches similar to those seen across East and South Africa such as prevention of mother-to-child transmission (PMTCT) and non-targeted community-based behaviour change programmes [5, 7].

Lessons learned from other contexts such as Southeast Asia and Latin America, where limited prevalence of HIV among average-risk reproductive-age adults also exists, require us to examine the epidemiology of the HIV epidemic in WCA [11, 29]. This systematic review aims to complete a historic, situational and epidemiological analysis of the burden of disease among key populations in 24 countries located in WCA.

## Methods

The US National Library of Medicine's MEDLINE database, one of the most comprehensive sources of healthcare information in the world, was searched using the PubMED interface to obtain biomedical markers for any of the three key populations: FSWs, MSM or PWID. The study objectives specified the need for epidemiologic studies that report biological endpoints (HIV prevalence) with defined methods; thus, it was decided a priori that MEDLINE would be sufficient. However, a sensitivity assessment was employed using the same search strategy to explore EBSCOhost CINAHL Plus, PsycINFO, Ovid, SocioFile and Popline, and no additional data points were obtained which met the defined inclusion and exclusion criteria. Google and Google Scholar were searched for contextual information and non-peer-reviewed literature. The Preferred Reporting Items for Systematic Reviews and Meta-Analyses (PRIMSA) guidelines were referenced for the development of the search protocol and study reporting structure [30, 31].

The medical subject headings (MeSH terms) for HIV and AIDS and key terms relating to “sex work,” “men who have sex with men” and “intravenous drug use” were cross-referenced with terms associated with 16 West African countries: the 15 countries of the Economic Community of West African States (ECOWAS: Benin, Burkina Faso, Cape Verde, Gambia, Ghana, Guinea (Conakry), Guinea-Bissau, Cote d'Ivoire, Liberia, Mali, Niger, Nigeria, Senegal, Sierra Leone and Togo) plus Mauritania. Eight Central African countries were included in the search: those in the Economic Community of Central African States (CEMAC: Cameroon, Chad, Equatorial Guinea, Central African Republic, Republic of Congo and Gabon), the Democratic Republic of Congo (DRC) and São Tomé and Príncipe. The search protocol was developed based on the objectives of this study and can be accessed as a Supplementary file with this manuscript.

The inclusion criteria for this study included reported HIV prevalence data for any of the three key populations, as well as clients of FSWs, in any of the 24 countries defined for this review. Publications were included if prevalence was listed in the article with sample size and sampling and HIV-testing methods described, regardless of the overall aim or topic of the study. Date of publication was not used as an inclusion criterion. Exclusion criteria included manuscripts not published in French, English or Spanish. Articles were downloaded and organized using Endnote (version X5), and data collection was finalized in April 2013.

### Screening and data abstraction

A title and abstract search protocol was utilized based on previously validated methods for systematic reviews [32]. At each step in the search protocol, the titles, abstracts and available data were appraised by two independent reviewers (LA and EP), and compiled and synthesized using standardized forms. During the title and abstract reviews, if either of the two reviewers considered the article relevant, it was included. Articles classified as relevant at the title review stage were downloaded for abstract and full-text evaluation. Data were independently extracted by two reviewers (LA and EP), then compared and consolidated for analysis.

Data, including sampling methods, HIV-1, HIV-2 and dual HIV-1 and -2 (HIV-1/2) infections with sample size and number of participants living with HIV, were detailed and coded by the two independent reviewers (LA and EP). Information was categorized by key population studied, sampling techniques, country or countries, sample size, number of study participants living with HIV and notes. Discrepancies in abstracted data from the two reviewers were assessed by a third reviewer independently evaluating the article (SB), as was the final consolidated database (CH).

## Results

**Figure 2:**
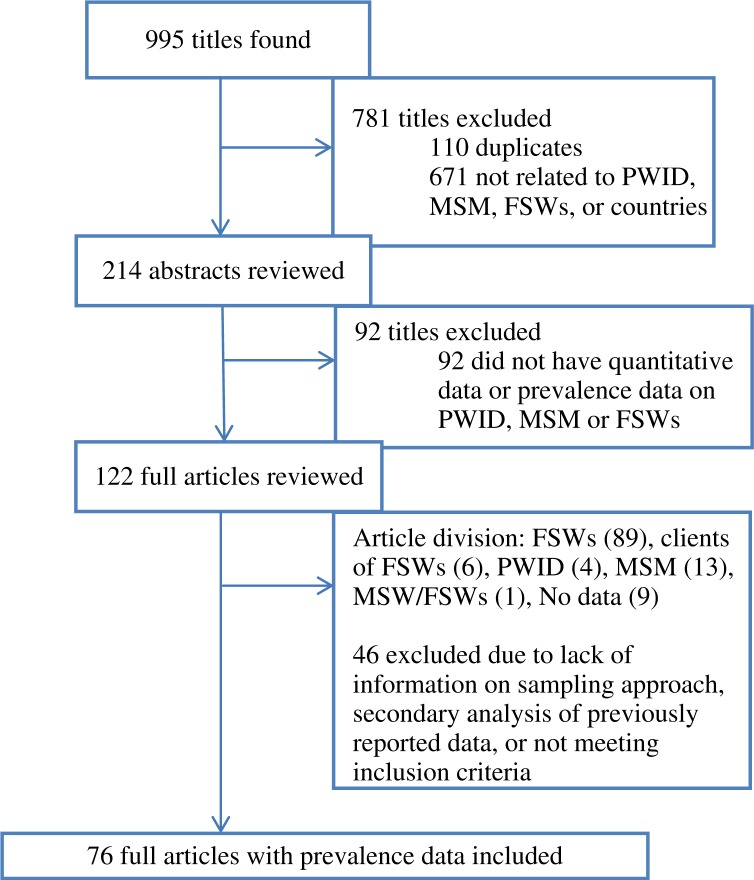
Flow chart of search findings and included studies.

Our search generated 995 citations, including 885 unique titles with dates of publication from 1987 to 2013 (Figure 2). Based on the inclusion criteria, 122 full articles were reviewed for data extraction, and 76 of these contained relevant data for at least one of the key populations defined. HIV prevalence data for at least one key population existed in 13 of the 24 countries included in the search (54.2%). Eleven of these countries were located in West Africa, and two countries were in Central Africa (DRC and Cameroon).

The majority of publications were assessments regarding FSWs (78.9%, 60/76), and another 10.5% (8/76) provided HIV prevalence data for their clients. One publication provided prevalence data for FSWs and well as clients of FSWs in Togo [33]. Thus, 90.8% (69/76) of the publications included in this study were related to FSWs, representing 41,270 FSWs across 13 countries and 5,986 clients of FSWs across 6 countries.

Two countries (Senegal and Nigeria) had published HIV prevalence data among MSM, and one seroprevalence study was conducted among male sex workers (MSWs) in Côte d'Ivoire, which was included in the MSM pooled data for analysis [34]. A total of six publications combined for the three countries were found for MSM (7.9%, 6/76), and one publication was available with HIV prevalence data for PWID, totalling 3,714 MSM from three countries and 1,459 PWID represented in the region.

Results presented in Table 1 show a pooled HIV prevalence for the relevant key population(s) in each country, the 95% confidence interval (CI), and the date(s) of the publications retrieved per country. We include both HIV-1 and HIV-2 infections in the pooled prevalence data for the country, and, when possible, we display the division of HIV-1, HIV-2 and HIV-1/2 infections. The far-left data column in Table 1 displays the overall HIV prevalence among reproductive-age adults (15–49) per country as reported by UNAIDS’ most recent country-level surveillance data [6].

**Table 1:** Pooled prevalence data for female sex workers (FSWs), clients of FSWs, MSM and PWID per country

Country	Year of publication(s)	Key population	Pooled HIV prevalence % (95% confidence interval)	Pooled HIV prevalence (sample size *N*=)	HIV-1 prevalence % (sample size *N*=)*	HIV-2 prevalence % (sample size *N*=)	HIV 1 and 2 prevalence % (Sample size *N*=)	HIV prevalence % Among adults 15–49**	Reference
Benin	1992, 1997, 2001, 2002, 2007, 2009, 2012	FSWs	45.8 (44.2–47.4)	3,885	41.8 (*N*=498)	3.2 (*N*=498)	11.2 (*N*=498)	1.1	[35–41]
	2000, 2007	Clients	6.7 (5.6–7.8)	1,996					[42,43]
Burkina Faso	1998, 2002	FSWs	45.8 (42.5–49.1)	873				1.0	[44,45]
Cameroon	1991, 1995, 1998, 1998, 2001, 2009	FSWs	23.6 (22.4–24. 8)	4,679	22.9 (*N*=2260)	0.04 (*N*=2260)		4.5	[41,46–50]
Cote d'Ivoire	1987, 1988, 1992, 1995, 1995, 1997, 1998, 2000, 2002, 2012	FSWs	57.3 (56.1–58.5)	7,014	40.0 (*N*=5204)	2.7 (*N*=5204)	21.1 (*N*=5204)	3.2	[17,29,66–73]
	2003	Clients	13.5 (10.2–16.8)	423					[74]
	2012	Male sex workers	50.0 (40.0–60.0)	96					[34]
DRC	1988, 1988, 1991, 1998, 2007	FSWs	26.3 (24.6–28.0)	2,518				1.1	[51–55]
Gambia	1991, 1991, 1993	FSWs	28.5 (25.0–32.0)	627	1.3 (*N*=627)	25.2 (*N*=627)	2.1 (*N*=627)	1.3	[56–58]
	1992	Clients	6.1 (4.1–8.1)	558					[59]
Ghana	2000, 2001, 2012	FSWs	60.4 (58.3–62.6)	1,982	46.7 (*N*=1348)	2.2 (*N*=1348)	6.7 (*N*=1348)	1.4	[40,60,61]
	2004	Clients	12.3 (9.4–15.2)	497					[62]
Guinea	2010, 2010, 2011	FSWs	36.9 (34.5–39.3)	1,577				1.7	[63–65]
Mali	1988, 1998	FSWs	42.1 (37.3–46.9)	406	35.8 (*N*=176)	3.9 (*N*=176)	6.2 (*N*=176)	0.9	[75, 76]
Niger	1994, 1998, 2006, 2006	FSWs	31.2 (28.4–34.1)	1,017	29.2 (*N*=767)	0.9 (*N*=529)	2.0 (*N*=767)	0.5	[77–80]
Nigeria	1989, 1993, 1993, 1993, 1998, 2002, 2008, 2011, 2012, 2012, 2013	FSWs	24.3 (23.5–25.1)	10,769	13.5 (*N*=2291)	1.9 (*N*=2041)	1.8 (*N*=610)	3.2	[81–91]
	2013	PWID	3.8 (2.8–4.8)	1,459					[92]
	2011, 2012, 2013	MSM	15.1 (13.7–16.5)	2,676					[93–95]
Senegal	1992, 1996, 1997, 2003, 2007, 2009	FSWs	19.0 (17.9–20.1)	4,612	7.6 (*N*=4008)	10.1 (*N*=4008)	1.1 (*N*=4008)	0.5	[96–101]
	1997, 2003	Clients	4.6 (3.6–5.7)	1,515					[102,103]
	2005, 2009, 2010	MSM	21.7 (19.1–24.3)	942	18.1 (*N*=442)	0.5 (*N*=442)	2.9 (*N*=442)		[104,105]
Togo	2009	FSWs	36.2 (33.6–38.8)	1,311				2.9	[18]
	2009	Clients	7.9 (6.2–9.6)	997					[18]

* Where available, the distribution of HIV1, HIV2 and dual HIV1/2 infections in the available study or pooled per country is listed.

** UNAIDS country prevalence data 2012 (6).

### Female sex workers and their clients

Behavioural and seroprevalence studies in FSWs were conducted consistently over time; however, there was a significant lull in published data between 2002 and 2007. When pooled, the overall HIV prevalence for FSWs in WCA was 34.9% (95% CI 34.4–35.4) (Table 2). In the five countries with six or more publications, pooled HIV prevalence was high: 57.3% (*N*=7,014) in Côte d'Ivoire, 24.3% (*N*=10,769) in Nigeria, 45.8% (*N*=3,885) in Benin, 23.6% (*N*=4,679) in Cameroon and 19.0% (*N =*4,612) in Senegal. The pooled prevalence found among clients of FSWs was 7.3% (95% CI 6.6–8.0) (Table 2). Six countries had at minimum of one study reporting prevalence data for this demographic, with publications as early as 1992 and as late as 2009 (Table 1).

**Table 2:** Pooled HIV prevalence data for female sex workers, clients of FSWs, MSM and PWID in West and Central Africa

Key population	Pooled HIV prevalence (%)	95% Confidence interval (%)	Pooled sample size, *N*=	*n*=Living with HIV
Female sex workers (FSWs)	34.9	34.4–35.4	41,270	14,388
Men who have sex with men (MSM)	17.7	16.5–18.9	3,714	656
People who inject drugs (PWID)	3.8	2.8–4.8	1,459	56
Clients of FSWs	7.3	6.6–8.0	5,986	435

### Men who have sex with men

While this review revealed a paucity of data for MSM, the pooled HIV prevalence in this review was 17.7% (95% CI 16.5–18.9) for MSM in WCA (Table 2). No studies included were published earlier than 2005, and all but one were published after 2010. Three relevant Nigerian studies showed a pooled prevalence of 15.1% compared to 3.2% in adults of reproductive age [6, 93–95, 106]. Senegal's pooled prevalence was 21.7% compared to 0.5% in the adults of reproductive age [6, 104, 105, 107]. The study conducted in Côte d'Ivoire among MSWs reported 50.0% prevalence among a sample of 96 men in Abidjan [34]. Snowball, convenience, purposive and respondent-driven sampling were the primary recruitment methods used to obtain these data.

### People who inject drugs

One study included directly sampled PWID. The study found a slightly higher prevalence of HIV at 3.8% (95% CI 2.8–4.8), compared to 3.2% in the general population in Nigeria [6, 92]. The sample was recruited through respondent-driven sampling and mainly compromised of men (>90%) [6, 92].

#### Limitations

This study was conducted as a systematic review to understand the prevalence of key populations in WCA and compare historical HIV prevalence to general-population statistics. Data were obtained from peer-reviewed literature, and while this ensures some quality control, we acknowledge that some relevant data that exist in grey literature and other programmatic data may have been overlooked. Programmatic data were not included in this review as it was not possible to implement a standardized assessment of the quality of the methods used and to ascertain an overview of research sampling and testing methods. However, the grey literature obtained through this review played a key role in the contextual analysis and discussion section of this study. Certain limitations also include the use of only English, French and Spanish, as other publications in other languages may have relevant data not captured in these inclusion criteria. The study among MSWs from Côte d'Ivoire was included in the overall analysis; however, the sampling method directly recruited these individuals from an established sex worker clinic, and thus HIV prevalence may be overestimated in this subpopulation. Also, while the authors noted that the majority of MSWs in the Abidjan area were MSM, they did not collect data on types of partner [34]. The contextual description from the authors is supported by evidence from other contexts where partners of MSWs are male [108, 109]. Concurrently, systematic review methods were applied; however, sensitivity analysis and meta-analyses were not utilized. While odds ratios or aggregated comparison data were not generated, the overall analysis provides an overview of HIV prevalence among key populations and details of the epidemiology of key populations since the debut of HIV research in this region.

## Discussion

Epidemiologic literature over the past 30 years has demonstrated a consistent and disproportionate burden of HIV among key populations in WCA. From the first published study in 1987 to the most recent in 2013, elevated levels of HIV among FSWs and their clients were consistently reported. In recent years, studies emerged to display an elevated burden of HIV among MSM within the region, although the number of studies in this subpopulation remains limited. Concurrently, there is nascent but growing evidence of the existence of PWID and, consequently, HIV infections in this subpopulation [92].

### HIV prevalence

The elevated HIV prevalence among MSM, FSWs and clients of FSWs is important based on the determinants of the HIV epidemic in WCA and even more broadly across SSA. Surveillance has shown that women carry the highest burden of HIV on the continent, with national-level statistics constantly reporting that women have a higher HIV prevalence and incidence than men [13, 110]. While programmes are designed to address the various risks associated with female HIV acquisition, the results of this study demonstrate that HIV risks are significantly higher among FSWs than women who do not sell sex in WCA. These results are substantiated by a systematic review of FSWs in low and middle-income countries, which showed that FSWs in SSA have a pooled prevalence of 36.9% (95% CI 36.2–37.5) with a background prevalence on the continent of 7.42% in females [15]. Globally, FSWs were 13.5 (95% CI 10.0–18.1) times more likely to be living with HIV than women of reproductive age [15]. Thus, the results of this review and the epidemiology of HIV among FSWs worldwide suggest that inclusion of and significant focus on these women and their clients are of importance to address these populations’ high HIV acquisition and transmission risks in WCA [11, 72, 81, 84, 96].

On a continent where women are disproportionately burdened with HIV, prevalence of 17.7% (95% CI 16.5–18.9) among MSM demonstrates a potentially concentrated epidemic in this key population. A prevalence of 7.3% (95% CI 6.6–8.0) in clients of FSWs is also elevated compared to the general male population of the region and calls into question prevention programmes targeting this population. For clients of FSWs, male acquisition is linked to behavioural risks associated with multiple sexual partners, limited condom use and concomitant infection of an STI, amongst other determinants that are specific to men who engage in transactional sex [16, 74, 111]. For MSM, recent research has emerged that displays the increased transmission of HIV during anal sex, as well as sexual role versatility during same-sex practices that increases individual HIV risks and drives transmission within sexual networks of MSM [21]. Thus, the acknowledgement of a heightened burden of disease in these populations is important for the design and implementation of specialized HIV prevention, treatment and care programmes regionally [16, 26].

The heightened HIV prevalence in the MSM community found in these results is not unexpected, although the lack of data in WCA is noteworthy. The high prevalence reported in this review is comparable to other continents, with research indicating that MSM around the world are 19 times more likely to be infected with HIV than their adult male counterparts [18]. Interestingly, same-sex practices in WCA were reported as early as 1996 in a published population-based review [112]. The authors noted that the cumulative number of positive cases had exponentially increased from 1985 to 1995, and the primary modes of transmission were heterosexual practices (73.0%), homosexual practices (0.8%) and mother-to-child transmission (6.0%) [112]. More recent behavioural studies equally noted homosexual behaviour in different demographic studies. In Nigeria, 11.4% of sexually active secondary school students reported same-sex practices, and 12.4% reported anal sex [113]. In two Ghanaian studies in 2006 and 2008, prison inmates reported same-sex practices or identified as homosexual at 30.8% and 29.5%, respectively [114, 115]. While sporadic reports of same-sex practices and elevated HIV prevalence have been reported in the region, there is limited targeted programme activity for these men [5, 116, 117]. What does exist is limited in scale, based on community-driven initiatives, and functioning in highly stigmatized settings [33, 117, 118].

While HIV prevalence in PWID was found to be relatively low, the Nigerian study provides two important details for programming in WCA. Firstly, while it has generally been assumed that PWID constitute a minimal presence in WCA, the study's ability to generate a sample size of 1459 through respondent-driven sampling indicates that this population does exist. Secondly, while HIV prevalence appears low, we know from other contexts that once HIV is introduced into this specific subpopulation, the possibilities of rapid spread and sustained transmission are great [119, 120]. Contextually, policy makers are becoming aware of an increase of drug trafficking in the region, with large quantities of drugs confiscated in the past few years, and the recent conflict in Mali ascribed mainly to this trade [121]. Further supporting evidence of this regional trade was found in behavioural data in prisoners. In the same Ghanaian study in 2006, 41% of inmates reported imprisonment for narcotics; 7.3% had used cocaine, 5.2% heroin and 4.2% phencyclidine [114]. In the 2008 Ghanaian prison study, 35% of 1336 prisoners reported ever injecting drugs [115]. As was seen in Afghanistan as well as Thailand, Cambodia and other Southeast Asian countries, migration, trafficking, drug use and the HIV epidemic are intrinsically linked [119, 120, 122]. Thus, this is an important population to identify and appropriately engage in WCA in the coming decade of HIV prevention and control.

### Historical perspective

This review also indicates that knowledge of HIV prevalence among key populations and the proportion of HIV infections attributable to key populations in WCA are not representative of new or changing dynamics of HIV transmission. In 1995, Djomand *et al*. noted that the male:female ratio of HIV infection in Côte d'Ivoire had declined over time and the gender ratio had shown females to be 4.8 times more likely to be infected than men in 1988, compared to 1.9 times more likely in 1991 [20]. The authors asserted that this decline displayed that the HIV epidemic was initially concentrated in a core group of FSWs and their male partners, and was potentially expanding in broader populations with less identifiable risk factors, similar to dynamics observed in other regions outside of SSA [20, 122–124]. In 2004, Côté *et al*. conducted a study of adult males (15–59) in Accra, Ghana, and attributed 84% of existing cases of HIV to sex work and other transactional sex [125]. A study in 2008 based on Demographic and Health Surveys across four countries in SSA, including Ghana, showed that men who ever paid for sex were more likely to have HIV than men who had not (odds ratio 1.89, 95% CI 1.57–2.28) [126].

In the capital city of Lomé, Togo, researchers estimated the attributable fraction of current HIV cases to sex work and other transactional sex was 32%, in contrast to only 2% of cases outside of Lomé [18]. Finally, recently in Nigeria, a modes of transmission study asserted that 23% of HIV infection was attributable to key populations, including 10% of new infections amongst MSM [93]. Despite high HIV prevalence among key populations and a high number of HIV in 2009, cases attributable to behaviours such as sex between men and sex work, systematic prevention and treatment programmes for key populations have not been implemented regionally [5]. While prevention programmes for FSWs and their clients have been noted in countries including Ghana, Côte d'Ivoire, Nigeria and Cameroon, the appropriate scale of these programmes and collected surveillance data are limited, and HIV prevention, treatment and care programming for key populations has failed to become a standard of best practices in the region [5].

### Economic and regional migration

Underlying dynamics of the epidemic indicate external, economic and urban-centred disparities have contributed to the complexity of the HIV epidemic in WCA over time. Domestic and international migration patterns were repeatedly reported and significantly mirrored economic crises and fluctuations in specific countries. For example, a study in Côte d'Ivoire documenting the FSW population that accessed health clinics between 1991 and 1998 noted a major shift in country of origin over time, with Nigerian women surveyed increasing from 2 to 56% between 1992 and 1998, and Ghanaian women decreasing from 82 to 9% in the same time period [29]. Other studies reported the migration of Ghanaian FSWs to other countries in the 1990s and asserted that the significant economic and political crises in the country at that time contributed to this migration [3, 35]. The proportion of Liberian FSWs included in the same Ivorian study was shown to have increased from 0% in 1992 to 15% in 1995, and then to have declined to 2% in 1998 [94]. This evolution reflects the first internal conflict experienced in Liberia in the 1990s (1989–1996) [127, 128]. In a study reviewing the spread of HIV among FSWs in four cities across SSA, researchers noted that Cameroonian FSWs were more likely to have migrated internally to urban centres, while in Benin 86% of the FSWs sampled were from another country [41]. The only MSM study to discuss countries of origin was the MSW study in Côte d'Ivoire. Of the 96 MSWs sampled in Abidjan, 7.3% (7/96) reported a different country of origin [34].

The importance of these findings is revealed in the HIV prevalence among immigrants in the various studies. Nigerian and Ghanaian FSWs in the 2002 Côte d'Ivoire study were 1.03 (0.47–2.23) and 3.69 (2.28–5.97) times more likely to be infected than their counterparts from Côte d'Ivoire, Liberia and other West African countries [106]. In Lomé, two-thirds of FSWs were immigrants, and Ghanaian FSWs were 1.68 (1.06–2.66) times more likely to be living with HIV [126]. Addressing the needs of migrating populations at risk for or living with HIV is crucial, as these populations have less access to health services, are less likely to understand their human rights, and are more likely to contract a disease [129]. These populations are also more likely to be mobile; thus, successful prevention services for immigrant or mobile FSWs could potentially have an important impact in the overall reduction of HIV transmission and acquisition in the region [129].

Concurrently, disparity of HIV prevalence per locality was repeatedly reported in the various studies reviewed. In the same study that cited higher HIV levels among Ghanaian FSWs in Lomé, the prevalence among Lomé FSWs in 2005 was reported at 45.4% compared to 17.7% in the rest of Togo [18]. In two studies in Benin, there was significant spatial variation in the burden of HIV. For example, a study conducted in six cities in 2005 showed prevalence for HIV as high as 48.2% in Parakou, compared to 16.4% in Abomey/Bohicon [36]. A similar study found HIV prevalence in Cotonou, Benin, among FSWs to be 38.5%, compared to a pooled prevalence in three other large cities of the country of 58.9% [35]. Therefore, from an HIV prevention perspective, cross-border initiatives, effective community-based networking and standardized programmes across urban and regional landscapes for key populations are relevant for the WCA region.

#### Ways forward

Our review makes clear that there is a significant gap in the literature and subsequent HIV programmes for key populations in WCA. This may be ascribed to the application of the HIV response model of SSA to WCA epidemiological and prevention approaches. However, as reports of high HIV prevalence among key populations have existed in the literature since 1987, it also calls into question the structural barriers to healthcare for populations that engage in these defined sexual behaviours in this region. As in other contexts, sex work and other transactional sex, same-sex practice, and drug use are either criminalized or highly stigmatized in this region, and public policies have ignored or generally declined to address the specific health needs of key populations [5, 130, 131]. Research has shown that macro-level policies that impede or deter health service delivery for key populations ultimately increase vulnerability to disease acquisition [23, 130, 132].

Data presented here provide a useful framework for HIV programming in the region. The inclusion of relevant sexual history and behavioural questions in large-scale surveillance surveys, such as DHS, may also be of benefit in obtaining a better overview of the epidemiology of key populations, both in WCA and worldwide. While the delivery of sensitive questions such as engagement in sex work, transactional sex, same-sex practices and drug use must be carefully administered (ideally not within the household setting), standardized national data collection would go far to inform country and regional policy development in WCA.

Subsequently, emerging data have shown that addressing the epidemic in key populations requires combined behavioural, biomedical and structural approaches [23, 133]. Limited condom use with regular sexual partners, unawareness of HIV status and co-infections with genital ulcerative diseases are contributing factors to heightened prevalence [10, 21, 116]. High prevalence among key populations concurrently has implications for prioritized biomedical interventions [21, 134].

While the knowledge that these populations have a higher risk for transmission and acquisition of HIV and other STIs is acknowledged, the method in which prevention and treatment programmes address these risks has yet to be firmly cemented in HIV prevention programming [13]. Researchers in the United States and elsewhere have demonstrated the importance of engaging populations in the continuum of HIV care – from undiagnosed cases to testing and diagnosis, followed by linkage to ongoing care and treatment [135]. The continuum of HIV care significantly reduces the viral load among people living with HIV and ultimately reduces transmission [135, 136]. In two recent studies in the United States, researchers found that due to advances in antiretroviral regimes, with 70–80% adherence to antiretroviral therapy (ART) by participants, durable viral suppression occurred in most individuals, lowering the possibility for onward HIV transmission [136, 137]. The findings indicate that the key to community viral suppression is early diagnosis of the disease, well-developed referral systems to clinical services, and care and support programmes that encourage adherence and access to treatment [136]. This approach has been shown to be effective in contexts with both high and low prevalence, and recent research from South Africa affirms that adequate ART coverage at the community level reduces incidence over time [138]. Thus, prevention programmes are beginning to show that distribution of prevention commodities and messages should be in concert with interventions that address the virology and biomedical aspects of care and treatment [135]. This is even more relevant for key populations who carry a significant burden of disease and ultimately are people living with HIV.

Structural factors acting at the macro- and meso-levels should not be ignored in WCA and are essential when building combination biomedical programmes [23, 139]. Criminalization and public policy neglect substantially inhibit key populations’ ability to access appropriate, life-sustaining and prevention-oriented health services. Policy-level gaps and community-level stigma must be addressed if programmes are to adequately confront the needs of these populations [140, 141]. Studies from other countries on the continent indicate the stigma experienced within their communities and at health services, significantly deters the uptake at clinical services for key populations [130, 142]. Public policies that adequately address the intricate health needs, reduce stigma and discrimination, and facilitate community and provider level HIV care and treatment delivery will highly benefit the overall control and prevention of HIV among key populations in WCA [23].

## Conclusions

This systematic review suggests that the concentrated HIV epidemic in WCA more closely resembles the epidemics in Southeast Asia and Latin America than those in the rest of SSA. This not only calls into question the response to the HIV epidemic in WCA but indicates that the region has an opportunity to adapt and develop region-specific prevention and treatment strategies. Targeted, cost-effective programmes that address not only behavioural but also biological and structural risk factors associated with HIV acquisition and transmission key populations should be engaged to reduce the onward spread of HIV. Prevention programmes should model strategies on appropriate programmes that reduce community viral loads, increase uptake of treatment among key populations and address the barriers to healthcare that exist in highly stigmatized settings. Ensuring that programmes rooted in community-based approaches address the continuum of HIV care, from diagnosis to viral suppression, will be a challenge but also a possible victory for HIV prevention and control in WCA.
